# Assessement of postoperative long-term survival quality and complications associated with radical antegrade modular pancreatosplenectomy and distal pancreatectomy: a meta-analysis and systematic review

**DOI:** 10.1186/s12893-019-0476-x

**Published:** 2019-01-28

**Authors:** Quanyu Zhou, Fengwei-Gao, Jie Gong, Qingyun Xie, Yu Liu, Qing Wang, Zehua Lei

**Affiliations:** Department of Hepatobiliary and Pancreatic Surgery, The People’s Hospital of Leshan City, Leshan, Sichuan 614000 People’s Republic of China

**Keywords:** Radical antegrade modular pancreatosplenectomy, Distal pancreatectomy, Pancreatic body/tail cancer, Meta-analysis

## Abstract

**Background:**

This study evaluated the perioperative complications and the long-term pancreatic survival outcomes in patients treated with radical antegrade modular pancreatosplenectomy (RAMPS) and distal pancreatectomy (DP).

**Method:**

We performed a computer search on the PubMed, Embase and Cochrane Library databases to retrieve the RCT or clinical trials comparing RAMPS and DP published before July of 2018. The quality of the included trials was assessed according to the inclusion and exclusion criteria by two researchers independently. The *RevMan* 5.3 software was used to extract and analyze the data.

**Result:**

A total of 5 retroprospective clinical trial articles comprising 285 patients were included in the study. The number of patients who underwent RAMPS were 135 and 150 for DP. There were significant differences (*P* < 0.05) in the operation time [WMD = − 63.93, 95% CI (− 68.86 ~ − 58.99), *P*<0.00001], and bleeding volume [WMD = − 184.62, 95% CI (− 211.88 ~ − 157.37), *P*<0.00001] between the two groups. However, no significant differences were observed between RAMPS and DP in terms of pancreatic fistula, postoperative complications, postoperative hospital stay, and mortality (*P*>0. 05). As for pathological examination, there were statistically significant differences between RAMPS and DP in the R0 resection rate [RR = 2.37, 95% CI (1.19 ~ 4.72), *P* = 0.01] and the number of lymph node excision [WMD = 7.08, 95% CI (4.59 ~ 9.58), *P*<0.000013]. The one-year overall survival rate was higher in RAMPS patients compared to DP patients [RR = 1.20, 95% CI (1.02 ~ 1.41), *P* = 0.02]. But there were no significant difference in postoperative recurrence [RR = 0.85, 95% CI (0.70 ~ 1.04), *P* = 0.13] between the two groups. Conclusion: RAMPS is an effective procedure for clinical application. Nevertheless, large, multicenter prospective randomized controlled trias are required to validate these findings.

**Conclusion:**

The RAMPS procedure was associated with good postoperative outcomes and overall survival, indicating that it is an effective procedure for clinical application. Large, multicenter prospective randomized controlled trials are needed to validate these findings.

**Electronic supplementary material:**

The online version of this article (10.1186/s12893-019-0476-x) contains supplementary material, which is available to authorized users.

## Background

Distal pancreatectomy (DP) is a major surgical procedure performed in patients with resectable pancreatic and tail tumors [1]. However, when a pancreatic tissue that has not been infiltrated by the tumor is resected, DP often leads to impaired secretory function of the pancreas and poor long-term quality of life of patients [2]. In 2003, Strasberg et al. introduced a new method of resection for pancreatic cancer (radical antegrade modular pancreatosplenectomy RAMPS) [3]. Unlike DP which is performed from left to right, RAMPS is a novel procedure that includes a horizontal dissection plane from right to left and a radical resection of regional lymph nodes based on dissection of the pancreas. RAMPS is now widely performed, and it produces negative tangential edges and favorable survival rates [4–6]. At present, the clinical outcomes of RAMPS have not been clearly defined. Most advanced studies on this topic are currently limited to single-center or small sample studies. Therefore, we conducted a systematic evaluation and meta-analysis to compare the results of distal pancreatectomy with those of radical anteroposterior modular pancreatoduodenectomy. The outcomes of this study are expected to guide further research and clinical selection of patients for surgery.

## Methods

### Literature search strategies

Computer retrieval of published randomized controlled trials or the rigorously designed clinical controlled trials comparing RAMPS and DP surgical procedures was performed in Pubmed, Embase, and Cochrane center databases before July 1, 2018. We searched for published data using the following terms: #1 research design [mh] OR clinical trials [mh] OR comparative study [mh] OR placebos [mh] OR multicenter study [pt] OR clinical trial [pt] OR random* [tiab] OR placebo*[tiab] OR clinical trial* [tiab] OR controlled clinical trial [pt] OR randomized controlled trial [pt] OR practice guideline [pt] OR feasibility studies [mh] OR clinical protocols [mh] OR single blind* [tiab] OR double blind* [tiab] OR triple blind* [tiab] OR treatment outcomes [mh] OR epidemiologic research design [mh] OR double-blind method [mh] OR pilot projects [mh]; #2“ radical antegrade modular pancreatosplenectomy “[MeSH]; #3 “Distal “OR “ Left “OR“ Far “OR“ Pancreatectomy “; #4 #1 AND((#2 OR #3). The systematic literature review and meta-analysis were performed in accordance with the recommendations of the Preferred Reporting Items for Systematic Reviews and Meta-Analyses (PRISMA) guidelines (Additional file 1) [7].

### Inclusion and exclusion criteria

Eligible trials were independently selected by two reviewers. Differences observed between the two reviewers were resolved through discussions with a third reviewer. Inclusion criteria were: (1) the included literature should contain the abstract and full text of the published literature before July 1, 2018; (2) literature design type: randomized controlled trial or clinical controlled trial with strict inclusion and exclusion criteria [8]; (3) to be included in the study, the general characteristics of the included patients should be explained; (4) the included patients were clearly diagnosed and (5) items to be included in the study for comparison: time of surgery, time of hospitalization, etc. should be provided. Exclusion criteria were:(1) the type of literature design is not clear, and the general situation of the included patients is not clear; (2) the included study language is limited to English; (3) data duplication; (4) the grouping description in the included literature is not clear, and some items are missing.

### Quality assessment

We chose to use the nine-star Newcastle Ottawa scale (NOS) to assess the methodological quality of case-control and cohort studies. The NOS scale included three evaluation criteria: patient selection (four stars), comparability (two stars) of the study group and outcome evaluation (three stars). Only studies with a score of 6 or above were considered to be qualitatively fit for a meta-analysis [9].

### Literature screening and data extraction

Literature screening: two researchers independently read and presented the literature for screening. Literature screening process was conducted according to PRISMA guidelines, and the whole process was conducted by the blinding method. First, the two researchers independently read the title and abstract of the literature obtained, and then excluded the literature that clearly did not meet the inclusion criteria. They then read the full text of the literature that appeared to meet the inclusion criteria to determine whether the literature met the inclusion criteria precisely. In the entire screening process, if there were disagreements, such as difficulties in determining whether a literature should be included or not, a resolution was arrived through discussion or by inclusion of a third researcher.

Data Extraction: After determining the literature to be included, the two researchers independently extracted data from the trials, including the follow-up of authors, publication date, number of cases, comparison indices between RAMPS and DP, such as morbidity, pancreatic fistula, bleeding rate, etc. The whole screening process was conducted in strict accordance with the systematic evaluation literature screening specification of the Cochrane collaboration group and the PRISMA standards for meta-analysis reporting [7].

### Statistical analysis

Statistical analysis was performed using *RevMan* (version 5.3; Cochrane Collaboration, Oxford, UK). Heterogeneity was assessed using the I2 statistical method. If there was no heterogeneity between studies (*I*^2^ ≤ 50%, *P* > 0.05), a fixed effect model was used; and if there was significant heterogeneity (*I*^2^>50%, *P*<0.05), we used a random effect model to combine the effects [10]. Counts and measures were analyzed by Relative Risk (RR) and weighted mean difference (WMD) with 95% confidence intervals (CI). *P* values of < 0.05 were considered statistically significant. The final result was represented by a forest diagram and the sensitivity analysis was used to test the robustness of the statistical results.

## Results

### Literature search and study characteristics

**Fig. 1:**
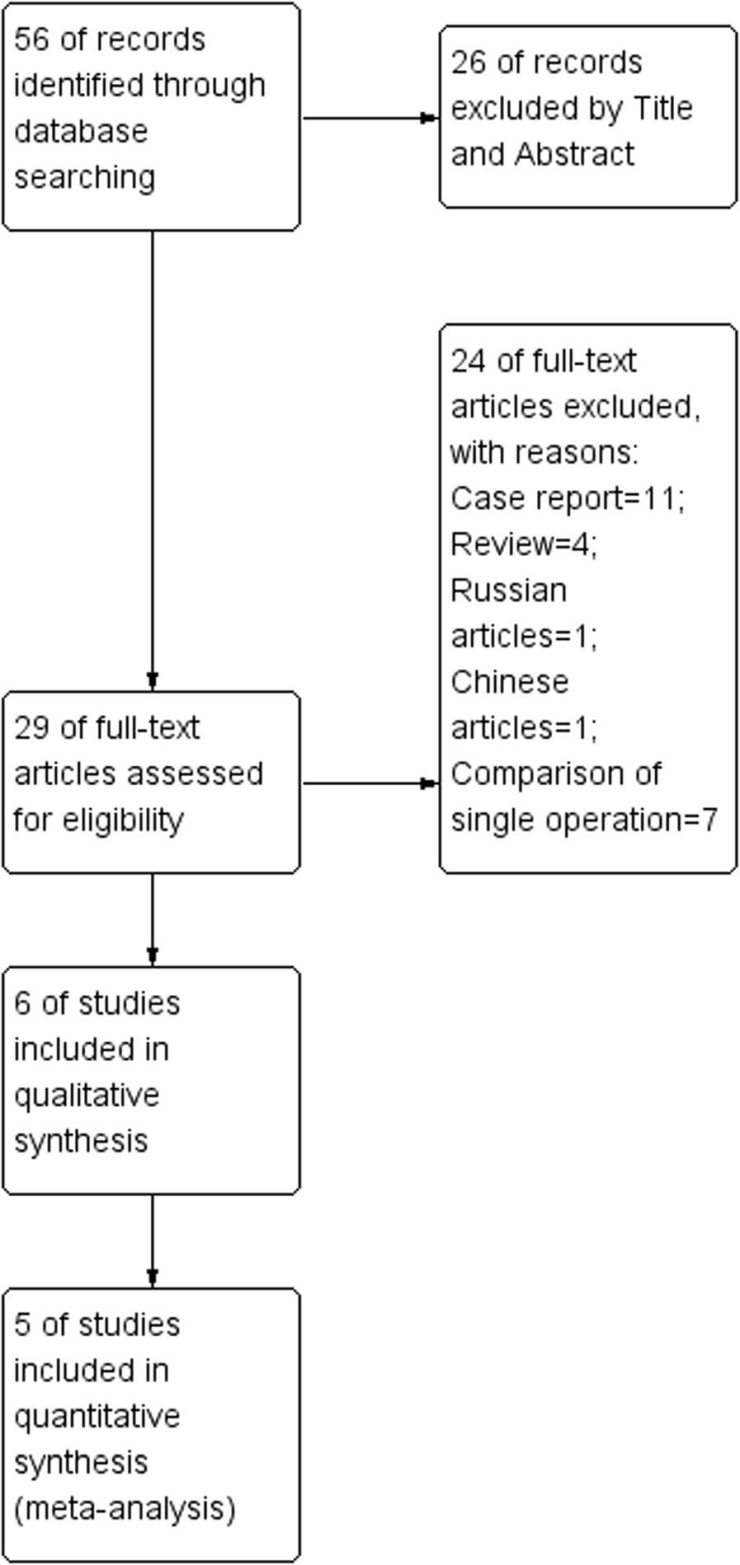
The flowchart of the literature selection process

Figure 1 shows the flowchart of the literature selection process. A total of 56 related studies were identified during the initial retrieval stage. We identified 26 studies based on the title and abstract. After careful evaluation, a total of 24 studies were excluded, as shown in Fig. 1. Finally, the remaining 5 case-control studies were included in the meta-analysis. The characteristics and quality of the included studies are summarized in Table 1 [11–15].

**Table 1:** Scale assessment of the quality of the studies

Author	Year	Location	Study	Group	Cases	Gender		Age	Follow-up time (month)	Newcastle – Ottawa scale Selection/comparability/Exposure = total score
						Male	Female			
PAUL TROTTMA [11].	2014	America	Retrospective	RAMPS	6	NA	NA	NA	NA	3/1/2 = 6
				DP	20	NA	NA	NA	NA	
Toshiya Abe [12]	2016	Japan	Retrospective	RAMPS	53	31	22	68.6 ± 10.7	267.3 ± 11.5	4/2/2 = 8
				DP	40	29	11	65.2 ± 8.6	339.4 ± 13.2	
Eun Young Kim [13]	2017	Korea	Retrospective	RAMPS	30	13	17	63.7 ± 8.2	33(8–97)	4/1/2 = 7
				DP	19	7	12	62.1 ± 8.5	33(8–97)	
Hyo Jun Park [14]	2014	Korea	Retrospective	RAMPS	38	23	15	62.17 (40–75)	18.2 (3.0–82.1)	4/2/3 = 9
				DP	54	35	19	61.25 (37–79)	15.7 (4.4–148.5)	
MARCO LATORRE [15]	2013	Italy	Retrospective	RAMPS	8	5	3	61	NA	4/1/2 = 7
				DP	17	11	6	60	NA	

### Data analysis

#### Surgical approach and postoperative pancreatic fistulas risk

**Fig. 2:**
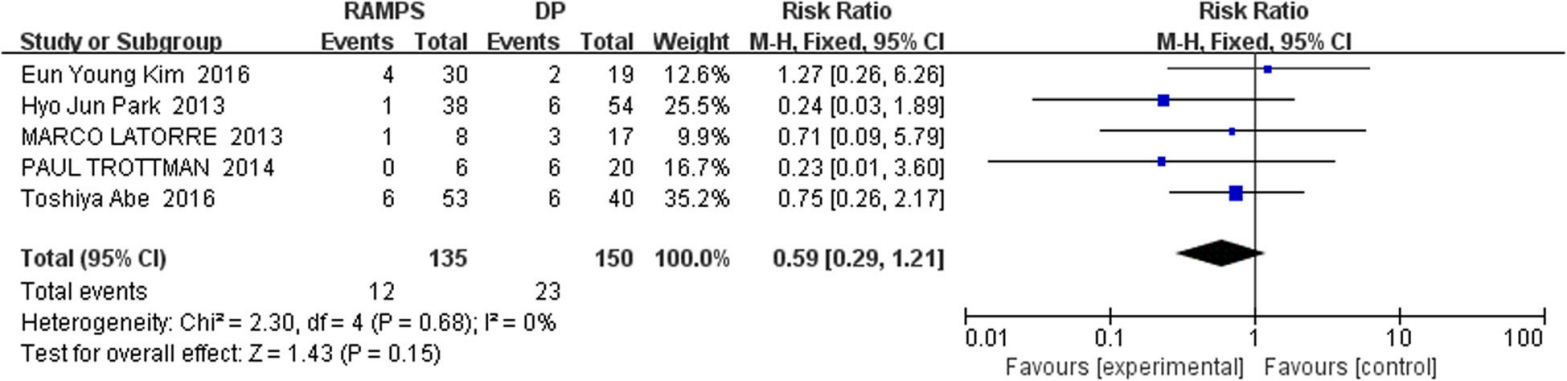
The forest diagram displaying the incidence of pancreatic fistula after RAMPS and DP

The forest diagram displaying the incidence of pancreatic fistula after RAMPS and DP is shown in Fig. 2. There were 5 retrospective studies, including 135 cases of RAMPS and 150 cases of DP. Low level of heterogeneity was found in the association results of the included studies (I^2^ = 0%, *P* for heterogeneity = 0.68). Based on the fixed-effect model analysis, the pooled RR for the difference between postoperative pancreatic fistula in RAMPS and DP cases was 0.59 (95% CI: 0.29~ 1.21). The pancreatic fistula rates were 8.8% (12/135) and 15.3% (23/150) in RAMPS group and DP group, respectively. No significant difference was observed in the occurrence of a postoperative pancreatic fistula between the two groups (*P* = 0.15).

#### Surgical approach and risk of other perioperative complications

A comparison of other complications between postoperative RAMPS and DP is shown in Table 2. The difference between RAMPS and DP in terms of operation time and blood loss was statistically significant (*P* < 0.05). There was no significant difference in postoperative complications, postoperative hospital stay, and mortality between RAMPS and DP (*P* > 0.05).

**Table 2:** Systematic reviews comparing postoperative RAMPS and DP complications

Factors	Included articles	RAMPS	DP	Heterogeneity test		RR/WMD	
				*I*^*2*^(%)	*P*	95% CI	*P*
Operation time	[10–12, 14]	97	96	98	<0.00001	−63.93(−68.86 ~ − 58.99)	<0.00001
Bleeding volume	[10–13]	127	133	88	0.0002	−184.62(−211.88 ~ − 157.37)	<0.00001
Postoperative complications	[10–13]	43	46	0	0.93	0.96 (1.55 ~1.65)	0.87
Postoperative hospital stay	[10–14]	135	150	64	0.04	−0.49(− 1.99 ~ 1.02)	0.53
Mortality(30-day)	[11–14]	129	130	NA	NA	0.65 (0.02 ~ 17.65)	0.80

#### Surgical approach and pathological examination risk

The results of the pathological examination of RAMPS and DP are shown in Table 3. The differences of R0 resection rate and lymph node resection number between the two groups were statistically significant (*P* < 0.05).

**Table 3:** Systematic reviews comparing RAMPS and DP pathological examination risk

Factors	Included articles	RAMPS	DP	Heterogeneity test		RR/WMD	
				*I*^*2*^(%)	*P*	95% CI	*P*
R0 resection	[10–14]	117	118	0	0.49	2.37 (1.19~4.72)	0.01
the number of lymph node	[10–12, 14]	97	96	0	0.85	7.08 (4.59~9.58)	<0.00001

#### Surgical approach and postoperative recurrence risk

**Fig. 3:**
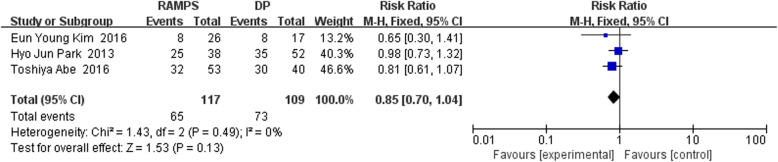
The forest diagram illustrating the incidence of postoperative recurrence in RAMPS and DP groups

The forest diagram illustrating the incidence of postoperative recurrence in RAMPS and DP groups is shown in Fig. 3. Three retrospective studies were included, with a total of 117 cases of RAMPS and 109 cases of DP. Assessement of the recurrence rate between RAMPS and DP was also described. The association results from each study were slightly heterogeneous (I^2^ = 0%, *P* for heterogeneity = 0.49). Based on the fixed-effect model analysis, the pooled RR for the difference between RAMPS and DP in terms of postoperative recurrence was 0.85 (95% CI: 0.70~1.04). The postoperative recurrence rates of RAMPS group and DP group were 55.5% (65/117) and 66.9% (73/109), respectively. There was no significant difference in postoperative recurrence between the two groups (*P* = 0.13).

#### Surgical approach and one-year overall survival risk

**Fig. 4:**
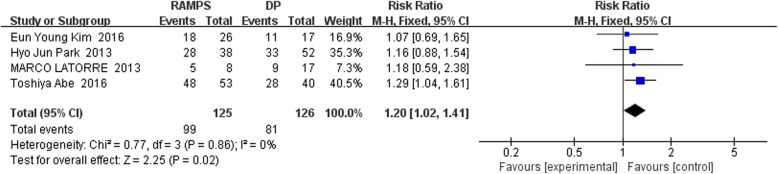
The forest diagram illustrating the one-year overall survival rate for RAMPS and DP

The forest diagram illustrating the one-year overall survival rate for RAMPS and DP is shown in Fig. 4. A total of 4 retrospective studies were included, including 125 cases of RAMPS and 126 cases of DP. There was low heterogeneity in the association results from each included trial (I^2^ = 0%, *P* for heterogeneity = 0.86). Using a fixed-effect model analysis, a pooled RR of 1.20 (95% CI: 1.02~1.41) was obtained for the difference between the overall annual survival rate in RAMPS and DP groups. The one-year total survival rates of RAMPS group and DP group were 78.2% (99/125) and 64.3% (81/126), respectively. The difference in terms of one-year overall survival between the two groups was statistically significant (*P* = 0.02).

## Discussion

Distal pancreatectomy (DP) was first proposed by the Mayo Clinic in 1913, which gradually became the standard surgical procedure for the body and tail of the pancreas [16]. However, it has been found that the number of intraoperative lymph nodes dissected and the long-term survival rate of patients are small. This has remained to be a challenge despite the fact that surgeons have perfected this procedure and are more skilled in other surgical techniques. To overcome the shortcomings of the traditional DP, in 2003, Strasberg performed a complete resection of the tumor while ensuring sufficient negative margins and the removal of lymph nodes in the region. This radical resection of the pancreatic body cancer from right to left combined with the spleen is known as the radical antegrade modular pancreatosplenectomy (RAMPS). Among the RAMPS patients reported by Strasbourg, there were 9 cases with R0 resection and 9 cases with average lymph node clearance without death or other complications [5]. By 2012, Strasberg and Fields had operated 80 RAMPS patients. The postoperative R0 excision rate was about 89%, and the 5-year survival rate increased from 26 to 35% [4]. Currently, there are few studies examining postoperative complications and postoperative long-term survival quality of life after RAMPS and DP. Therefore, we systematically evaluated the differences in postoperative complications and postoperative long-term survival rate between RAMPS and DP to guide further research and clinical selection of patients for surgery.

The data reviewed showed that there were significant differences in operation time and intraoperative blood loss between RAMPS and DP (*P* < 0.05). Postoperative pancreatic fistula, postoperative complications, postoperative hospital stay, and mortality were not statistically significant (*P* > 0.05). The operation time of patients with RAMPS was relatively long, mainly because of pancreas and spleen movement, venous disconnection, local lymph node dissection, and posterior peritoneal tissue cleaning. Intraoperative blood loss was lower in RAMPS compared to DP. This may be due to the need to separate the pancreatic neck first, followed by a right to left surgery to effectively expose the surgical wound. Regarding postoperative complications, this study found no significant differences in the incidence of postoperative complications between the two groups. Pancreatic fistula is considered to be the most important postoperative complication and is a potentially serious, life-threatening event that can extend hospitalization and increase costs. Regardless of the technique used to cut and close the pancreatic stump, the reported occurrence rate of pancreatic fistula varies greatly from 2% to more than 20% [17]. Although RAMPS is more difficult to perform than conventional surgery, it requires more retroperitoneal dissection, vascular nebulization, and lymph node dissection. However, the incidence of complications (pancreatic fistula) was not significantly different between RAMPS and DP. This proves that RAMPS is a safe and effective procedure.

The efficacy of RAMPS was mainly evaluated by pathological examination and long-term survival of patients after surgery. Pathological examination was assessed in terms of the rate of R0 excision and the number of lymph nodes resected. The results indicated that both the postoperative R0 resection rate and the number of lymph nodes resected were lower in DP group compared to RAMPS group, and the difference was statistically significant (*P* < 0.05). We postulate that these findings may be due to the following reasons: First, RAMPS is based on the anatomical architecture of the posterior pancreatic peritoneal fusion fascia (Gerota fascia, Treitz fascia, and Toldt fascia). Using Kocher approaches, the inferior vena cava and left renal vein along the Treitz fascia level; behind the Gerota fascia, the left renal vein, the renal capsule, and the left adrenal gland, are separated to achieve a complete resection of the nerve fiber connective tissue of the tail, spleen, and lymph nodes, enhancing the rate of R0 resection of the posterior peritoneum. In addition, RAMPS includes lymph node dissection based on the pattern of pancreatic lymphatic drainage to expose and clean all lymph nodes, which is difficult to achieve in traditional DP surgery.

Although there was no significant difference between RAMPS and DP in postoperative tumor recurrence (*P* > 0.05), there was a significant difference in long-term survival between the two groups (*P*<0.05). Studies have shown that RAMPS can prolong postoperative survival time compared with DP. In theory, RAMPS may reduce the local recurrence rate of tumors but not influence the systemic recurrence. However, based on our data, it is not easy to determine whether the local recurrence site is within the pancreatic stump, tumor bed or regional lymph node, making it difficult to determine the actual effect of RAMPS on tumor recurrence. We suggest that, when RAMPS relapse occurs, the time, location, frequency of recurrence and its effect on prognosis of the disease should be carefully monitored in future studies.

Our systematic review summarizes most of the available evidence in this regard. However, it has the following limitations: (1) although the included studies were of high-quality retrospective case-control studies, there was some deviations in patient selection; (2) the lack of randomness and retrospective studies introduces structural deviations that may lead to inaccurate data interpretation; (3) the postoperative management is different among different surgical centers, and the postoperative complications in patients are equally different; (4) the total number of cases in this study was small and there were regional differences. Therefore, large sample, multicenter, and standardized prospective randomized controlled trials are needed. The differences in postoperative complications and long-term survival were further discussed.

Our systematic review summarizes most of the available evidence in this field. However, it has the following limitations: (1) although the included articles were of high-quality retrospective case-control studies, some deviations in patient selection were apparent; (2) the lack of randomness and the retrospective nature of this study carries some structural deviations that may lead to inaccurate data analysis; (3) the postoperative management approaches were different among the surgical centers, and thus the postoperative complications in patients are expected to be different; (4) the total number of cases included in this study was relatively small and there were regional differences. Therefore, large sample, multicenter, and standardized prospective randomized controlled trials are needed to validate these findings. The differences in postoperative complications and long-term survival should be further evaluated.

## Conclusions

Application of RAMPS procedure for pancreatic body and tail tumors produces favorable outcomes and survival rates without increasing complications compared with the Distal pancreatectomy (DP). It decreases the occurrence of pancreatic fistula, which is a fatal complication associated with high morbidity and prolonged hospital stay after surgery. But it needs to strictly grasp the indications for surgery in the clinical.

## Additional file

Additional file 1: **Appendix 1.** The Preferred Reporting Items for Systematic Reviews and Meta-Analyses (PRISMA) 2009 Checklist. **Appendix 2.** The Risk of bias in the included retrospective cohort studies (by the Newcastle–Ottawa quality assessment tool). **Appendix 3.** The forest map of the incidence of Operation time between RAMPS and DP. **Appendix 4.** The forest map of the incidence of Bleeding volume between RAMPS and DP. **Appendix 5.** The forest map of the incidence of Postoperative complications between RAMPS and DP. **Appendix 6.** The forest map of the incidence of hospital stay between RAMPS and DP. **Appendix 7.** The forest map of the incidence of Mortality(30-day) between RAMPS and DP. **Appendix 8.** The forest map of the incidence of R0 resection between RAMPS and DP. **Appendix 9.** The forest map of the incidence of the number of lymph node between RAMPS and DP. **Appendix 10**. Search strategy. (DOCX 108 kb)

## Abbreviations

DP: Distal pancreatectomy
NOS: Newcastle-Ottawa Scale
RAMPS: Radical antegrade modular pancreatosplenectomy
RCTs: Randomized controlled trials
