# Supplementary material for: Assessement of postoperative long-term survival quality and complications associated with radical antegrade modular pancreatosplenectomy and distal pancreatectomy: a meta-analysis and systematic review
Source: BMC Surg. 2019 Jan 28;19:12. doi: 10.1186/s12893-019-0476-x (PMC6348671; doi:10.1186/s12893-019-0476-x)
Supplement: Supplementary file 1 — Appendix 1. The Preferred Reporting Items for Systematic Reviews and Meta-Analyses (PRISMA) 2009 Checklist. Appendix 2. The Risk of bias in the included retrospective cohort studies (by the Newcastle–Ottawa quality assessment tool). Appendix 3. The forest map of the incidence of Operation time between RAMPS and DP. Appendix 4. The forest map of the incidence of Bleeding volume between RAMPS and DP. Appendix 5. The forest map of the incidence of Postoperative complications between RAMPS and DP. Appendix 6. The forest map of the incidence of hospital stay between RAMPS and DP. Appendix 7. The forest map of the incidence of Mortality(30-day) between RAMPS and DP. Appendix 8. The forest map of the incidence of R0 resection between RAMPS and DP. Appendix 9. The forest map of the incidence of the number of lymph node between RAMPS and DP. Appendix 10. Search strategy. (DOCX 108 kb) [file 12893_2019_476_MOESM1_ESM.docx]

The comparison of radical antegrade modular pancreatosplenectomy versus distal pancreatectomy in complications and postoperative long term survival quality: a Meta analysis and systematic review

Additional file

Appendix file 1 the Preferred Reporting Items for Systematic Reviews and Meta-Analyses (PRISMA) 2009 Checklist

Appendix file 2 the Risk of bias in the included retrospective cohort studies (by the Newcastle–Ottawa quality assessment tool)

Appendix file 3 the forest map of the incidence of Operation time between RAMPS and DP

Appendix file 4 the forest map of the incidence of Bleeding volume between RAMPS and DP

Appendix file 5 the forest map of the incidence of Postoperative complications between RAMPS and DP

Appendix file 6 the forest map of the incidence of hospital stay between RAMPS and DP

Appendix file 7 the forest map of the incidence of Mortality(30-day) between RAMPS and DP

Appendix file 8 the forest map of the incidence of R0 resection between RAMPS and DP

Appendix file 9 the forest map of the incidence of the number of lymph node between RAMPS and DP

Additional file 10: Search strategy

Appendix 1 the Preferred Reporting Items for Systematic Reviews and Meta-Analyses (PRISMA) 2009 Checklist


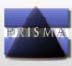
**PRISMA 2009 Checklist**

| **Section/topic** | **#** | **Checklist item** | **Reported on page #** |
| --- | --- | --- | --- |
| **TITLE** | | |  |
| Title | 1 | Identify the report as a systematic review, meta-analysis, or both. | 1 |
| **ABSTRACT** | | |  |
| Structured summary | 2 | Provide a structured summary including, as applicable: background; objectives; data sources; study eligibility criteria, participants, and interventions; study appraisal and synthesis methods; results; limitations; conclusions and implications of key findings; systematic review registration number. | 1 |
| **INTRODUCTION** | | |  |
| Rationale | 3 | Describe the rationale for the review in the context of what is already known. | 2 |
| Objectives | 4 | Provide an explicit statement of questions being addressed with reference to participants, interventions, comparisons, outcomes, and study design (PICOS). | 2 |
| **METHODS** | | |  |
| Protocol and registration | 5 | Indicate if a review protocol exists, if and where it can be accessed (e.g., Web address), and, if available, provide registration information including registration number. | 3 |
| Eligibility criteria | 6 | Specify study characteristics (e.g., PICOS, length of follow-up) and report characteristics (e.g., years considered, language, publication status) used as criteria for eligibility, giving rationale. | 3 |
| Information sources | 7 | Describe all information sources (e.g., databases with dates of coverage, contact with study authors to identify additional studies) in the search and date last searched. | 3 |
| Search | 8 | Present full electronic search strategy for at least one database, including any limits used, such that it could be repeated. | 3 |
| Study selection | 9 | State the process for selecting studies (i.e., screening, eligibility, included in systematic review, and, if applicable, included in the meta-analysis). | 3 |
| Data collection process | 10 | Describe method of data extraction from reports (e.g., piloted forms, independently, in duplicate) and any processes for obtaining and confirming data from investigators. | 4 |
| Data items | 11 | List and define all variables for which data were sought (e.g., PICOS, funding sources) and any assumptions and simplifications made. | 4 |
| Risk of bias in individual studies | 12 | Describe methods used for assessing risk of bias of individual studies (including specification of whether this was done at the study or outcome level), and how this information is to be used in any data synthesis. | 4 |
| Summary measures | 13 | State the principal summary measures (e.g., risk ratio, difference in means). | 4 |
| Synthesis of results | 14 | Describe the methods of handling data and combining results of studies, if done, including measures of consistency (e.g., I^2^) for each meta-analysis. | 4 |

Page 1 of 2

| **Section/topic** | **#** | **Checklist item** | **Reported on page #** |
| --- | --- | --- | --- |
| Risk of bias across studies | 15 | Specify any assessment of risk of bias that may affect the cumulative evidence (e.g., publication bias, selective reporting within studies). | 4 |
| Additional analyses | 16 | Describe methods of additional analyses (e.g., sensitivity or subgroup analyses, meta-regression), if done, indicating which were pre-specified. | 4 |
| **RESULTS** | | |  |
| Study selection | 17 | Give numbers of studies screened, assessed for eligibility, and included in the review, with reasons for exclusions at each stage, ideally with a flow diagram. | 5,Fig1 |
| Study characteristics | 18 | For each study, present characteristics for which data were extracted (e.g., study size, PICOS, follow-up period) and provide the citations. | 5, Table 1 |
| Risk of bias within studies | 19 | Present data on risk of bias of each study and, if available, any outcome level assessment (see item 12). | 5-7 |
| Results of individual studies | 20 | For all outcomes considered (benefits or harms), present, for each study: (a) simple summary data for each intervention group (b) effect estimates and confidence intervals, ideally with a forest plot. | 5-7 |
| Synthesis of results | 21 | Present results of each meta-analysis done, including confidence intervals and measures of consistency. | 5-7 |
| Risk of bias across studies | 22 | Present results of any assessment of risk of bias across studies (see Item 15). | 5-7 |
| Additional analysis | 23 | Give results of additional analyses, if done (e.g., sensitivity or subgroup analyses, meta-regression [see Item 16]). | 5-7 |
| **DISCUSSION** | | |  |
| Summary of evidence | 24 | Summarize the main findings including the strength of evidence for each main outcome; consider their relevance to key groups (e.g., healthcare providers, users, and policy makers). | 7 |
| Limitations | 25 | Discuss limitations at study and outcome level (e.g., risk of bias), and at review-level (e.g., incomplete retrieval of identified research, reporting bias). | 10 |
| Conclusions | 26 | Provide a general interpretation of the results in the context of other evidence, and implications for future research. | 11 |
| **FUNDING** | | |  |
| Funding | 27 | Describe sources of funding for the systematic review and other support (e.g., supply of data); role of funders for the systematic review. | No funder |

*From:*  Moher D, Liberati A, Tetzlaff J, Altman DG, The PRISMA Group (2009). Preferred Reporting Items for Systematic Reviews and Meta-Analyses: The PRISMA Statement. PLoS Med 6(7): e1000097. doi:10.1371/journal.pmed1000097

For more information, visit: **www.prisma-statement.org**.

Appendix 2 Risk of bias in the included retrospective cohort studies (by the Newcastle–Ottawa quality assessment tool)

Supplemental NOS for Cohort Studies

|  | **Representativeness of the exposed cohort** | **Selection of the non-exposed cohort** | **Ascertainment of exposure** | **Demonstration that outcome of interest was not present at start of study** | **Comparability of cohorts on the basis of the design or analysis^b^** | **Assessment of outcome** | **Was follow-up long enough for outcome to occur** | **Adequacy of follow-up of cohorts** | **Total quality scores** |
| --- | --- | --- | --- | --- | --- | --- | --- | --- | --- |
| **MARCO LATORRE**  (2013) | * | - | * | * | - | * | * | * | 7 |
| **Hyo Jun Park**  (2014) | * | * | * | * | ** | * | * | * | 9 |
| **PAUL TROTTMA**  (2014) | * | * | * | * | * | * | - | - | 6 |
| **Toshiya Abe**  (2016) | * | * | * | * | ** | * | - | * | 8 |
| **Eun Young Kim**  (2017) | * | * | - | * | ** | * | * | * | 7 |

a A study can be awarded a maximum of one star for each numbered item except for the item Control for important factor or additional factor.

b A maximum of two stars can be awarded for Control for important factor or additional factor.

Appendix 3 the forest map of the incidence of Operation time between RAMPS and DP


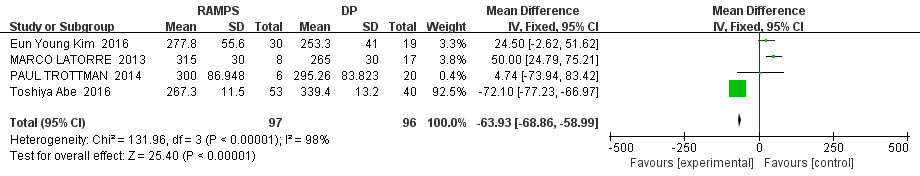


Appendix4 the forest map of the incidence of Bleeding volume between RAMPS and DP


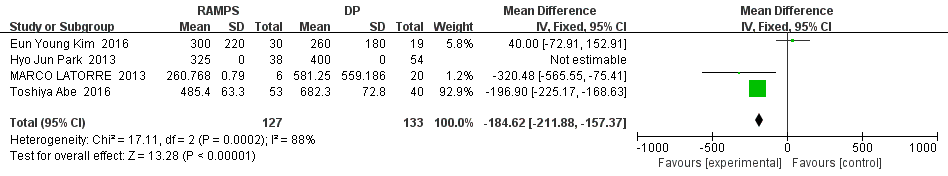


Appendix5 the forest map of the incidence of Postoperative complications between RAMPS and DP


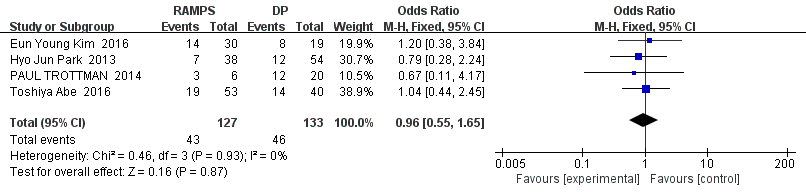


Appendix6 the forest map of the incidence of hospital stay between RAMPS and DP


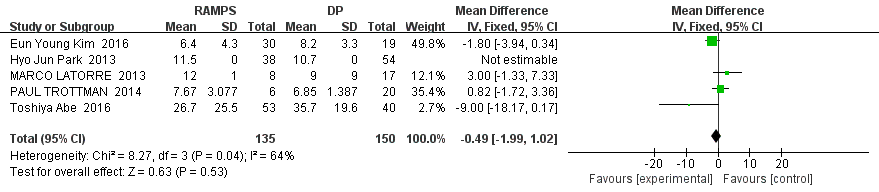


Appendix7 the forest map of the incidence of Mortality(30-day) between RAMPS and DP


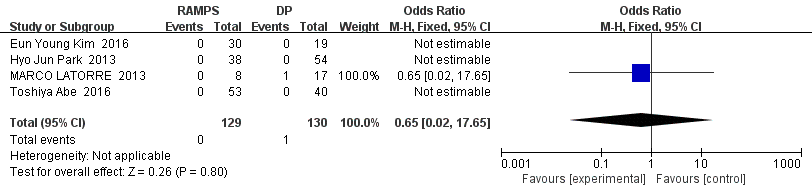


Appendix8 the forest map of the incidence of R0 resection between RAMPS and DP


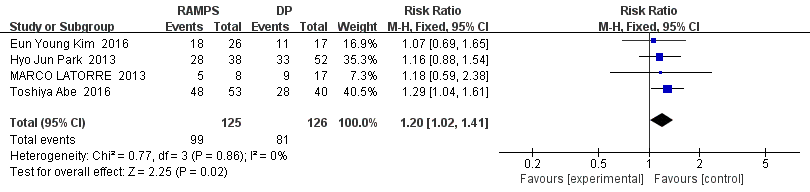


Appendix9 the forest map of the incidence of the number of lymph node between RAMPS and DP


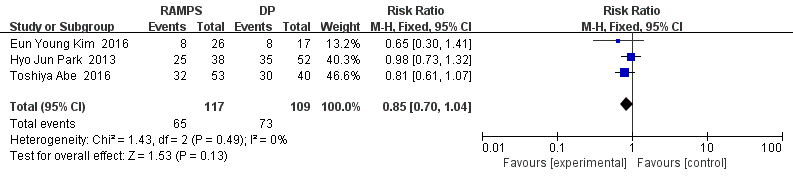


Appendix10: Search strategy

A review of the literature was performed in July of 2018 to produce a bibliography of articles, abstracts, and research reports in which comparing radical antegrade modular pancreatosplenectomy versus distal pancreatectomy was studied in a randomized controlled trial. The following databases were searched: Pubmed, Embase and Cochrane Library to identify relevant studies. Studies were retrieved regardless of language. Electronic searches were supplemented by hand searching reference lists and reviews.

MEDLINE is the premier biomedical bibliographic database, produced by the National Library of Medicine (NLM). NLM indexes over 4600 worldwide life science journals. MEDLINE includes over 12 million references from 1966 to the present. Indexers assign Medical Subject Headings (MeSHs), NLM’s controlled thesaurus of descriptors. Most of the references starting in 1975 also contain an abstract. MEDLINE was searched using the PubMed search interface.

The following search strategy was used:

#1 research design [mh] OR clinical trials [mh] OR comparative study [mh] OR placebos [mh] OR multicenter study [pt] OR clinical trial [pt] OR random* [tiab] OR placebo*[tiab] OR clinical trial* [tiab] OR controlled clinical trial [pt] OR randomized controlled trial [pt] OR practice guideline [pt] OR feasibility studies [mh] OR clinical protocols [mh] OR single blind* [tiab] OR double blind* [tiab] OR triple blind* [tiab] OR treatment outcomes [mh] OR epidemiologic research design [mh] OR double blind method [mh] OR pilot projects [mh]

#2 " radical antegrade modular pancreatosplenectomy "[MeSH]

#3 " Distal "OR " Left "OR " Far "OR" Pancreatectomy "

#4 #1AND((#2 OR #3)

The Cochrane Central Register of Controlled Trials (CCTR) is a component of the Cochrane Evidence-Based Medicine Reviews Collection. It is a bibliographic database of definitive controlled trials identified by the contributors to the Cochrane Collaboration. Cochrane groups and other organizations contribute their specialized registers, and together with references to clinical trials identified in MEDLINE and EMBASE, form the CCTR database. CCTR was searched using the following search strategy:

#1 [SUTURE TECHNIQUES explode tree 1 (MeSH)](http://212.49.218.200/newgenMB/ASP/srchResults.asp?histNo=1)

#2 [INTESTINES explode tree 1 (MeSH)](http://212.49.218.200/newgenMB/ASP/srchResults.asp?histNo=2)

#3 [(#1 and #2)](http://212.49.218.200/newgenMB/ASP/srchResults.asp?histNo=3)

The Excerpta Medica Database (EMBASE) is a biomedical and pharmacological resource providing access to the most up-to-date information about medical and drug-related subjects. It covers the literature from 1974 to the present. EMBASE provides access to periodical articles from more than 3,700 primary journals from approximately 70 countries. EMBASE was searched using the following search strategy:

1. radical antegrade modular pancreatosplenectomy? Or Distal pancreatectomy?
2. random? And trial? ?
3. 1 and 2

We also searched the reference lists from the primary studies and review articles, sought help from experts in the field. The subsequent criteria for selection of trials to be included in the meta-analysis are described in the Methods section, and the flowchart of trials is presented in Figure 1.
